# The Bewildering Effect of AMPK Activators in Alzheimer's Disease: Review of the Current Evidence

**DOI:** 10.1155/2020/9895121

**Published:** 2020-02-18

**Authors:** Brhane Teklebrhan Assefa, Gebrehiwot Gebremedhin Tafere, Dawit Zewdu Wondafrash, Meles Tekie Gidey

**Affiliations:** ^1^Department of Pharmacology and Toxicology, School of Pharmacy, College of Health Sciences, Mekelle University, Mekelle, Ethiopia; ^2^Pharmacoepidemiology and Social Pharmacy Unit, School of Pharmacy, College of Health Sciences, Mekelle University, Mekelle, Ethiopia

## Abstract

Alzheimer's disease is a multifactorial neurodegenerative disease characterized by progressive cognitive dysfunction. It is the most common form of dementia. The pathologic hallmarks of the disease include extracellular amyloid plaque, intracellular neurofibrillary tangles, and oxidative stress, to mention some of them. Despite remarkable progress in the understanding of the pathogenesis of the disease, drugs for cure or disease-modifying therapy remain somewhere in the distance. From recent time, the signaling molecule AMPK is gaining enormous attention in the AD drug research. AMPK is a master regulator of cellular energy metabolism, and recent pieces of evidence show that perturbation of its function is highly ascribed in the pathology of AD. Several drugs are known to activate AMPK, but their effect in AD remains to be controversial. In this review, the current shreds of evidence on the effect of AMPK activators in A*β* accumulation, tau aggregation, and oxidative stress are addressed. Positive and negative effects are reported with regard to A*β* and tauopathy but only positive in oxidative stress. We also tried to dissect the molecular interplays where the bewildering effects arise from.

## 1. Introduction

Alzheimer's disease (AD) is a progressive neurodegenerative disease clinically characterized by cognitive, functional, and behavioral alterations. It is also characterized by histopathological, molecular, and biochemical abnormalities such as cell loss, intracellular accumulation of neurofibrillary tangles, dystrophic neurites, amyloid-*β* deposits, deranged energy metabolism, mitochondrial dysfunction, chronic oxidative stress, and DNA damage [1]. Of these pathologic changes, intracellular accumulation of neurofibrillary tangles is both spatially and temporally closely associated with neuronal degeneration and cognitive symptoms [2].

There are two types of AD: familial and sporadic. The familial one accounts for around 1% of all AD cases and occurs at the age below 60 years. Causative variants have been identified in genes encoding amyloid precursor protein (APP), presenilin-1, and presenilin-2. The sporadic type is the more common type of AD and usually is a late-onset AD occurring after the age of 60 years [3]. Collectively, they are regarded as global crisis affecting the aging population and the society as a whole. In 2010, around 35.6 million people lived with dementia worldwide, and in 2015, it was estimated that 46.8 million people suffer from dementia. This is expected to project up to 131.5 million in the next 30 years, doubling approximately every 20 years [4]. Out of this, up to 75% is Alzheimer's dementia. This shows that the prevalence of AD is steeply increasing and it is expected to pose a huge challenge to public health and the elderly care system globally in the future. This increasing prevalence and incidence can be attributed to the aging population phenomenon [5, 6]. The prevalence of AD increases exponentially with age. At the age of 60–64 years, the prevalence of AD is 2% whereas for the age group 80–84 the prevalence is up to 13% [7, 8].

AD has several impacts on the patients, families, communities, and healthcare systems. It is the sixth leading cause of the total deaths and fifth leading cause of death among people aged ≥65 years in the United States [9]. Currently, there is no treatment that reverses or slows the progress of the disease [3]. Till a recent time, it has been thought as an inevitable effect of aging. However, several lines of evidence are flourishing which show that AD is associated with several modifiable risk factors. For example, a study in 2017 which summarized the latest shreds of evidence on different risk factors showed that around 35% of AD is linked to potentially modifiable factors [10, 11]. This shows there still is a room to find the pharmacological intervention to promote healthy brain aging.

Increasing epidemiological and biological evidence shows that there is a strong correlation between AD and type 2 diabetes mellitus (T2DM) [12]. AD and T2DM have several commonalities in their pathologies such as insulin resistance, disturbed glucose and lipid metabolism, inflammation, and oxidative stress [13–15]. Several series studies also showed a strong correlation between insulin signaling and amyloid-*β* metabolism and tau phosphorylation. Insulin is found to inhibit amyloid-*β* generation and to prevent hyperphosphorylation of tau protein (the main pathologic hallmark of AD) [16, 17]. In contrast, insulin resistance and insulin deficiency are associated with the increased extracellular amyloid-*β* accumulation and intracellular hyperphosphorylated tau [1, 18–20]. Postmortem studies showed decreased synaptic plasticity in T2DM patients [21], and other studies showed insulin administration to improve synaptic formation [22, 23] and cognitive performance [24–26]. All these findings indicate a robust correlation between AD and T2DM. Even it was once proposed that AD may be brain type DM “type 3 DM” [27, 28]. In this regard, it is worth discussing the most important common molecule involved in the pathogenesis of both AD and T2DM.

Recent studies show that one of the common pathways for AD and T2DM involves AMP-activated protein kinase (AMPK) [29, 30]. In an attempt to investigate the molecular basis of the comorbidity in AD and T2DM, Caberlotto et al. applied systems biology approach and showed that autophagy is at the center of the comorbidity [1]. A wealth of recent studies also showed that AMPK is the principal player in both energy metabolism and autophagy [31–33]. This opens a new direction in the discovery of disease-modifying drugs. In view of this, AMPK can be a viable drug target for the treatment of AD. Though controversial, several recent studies showed the potential druggability of this particular enzyme. Therefore, the aim of this review is to summarize the current evidence regarding the potential use of AMPK activators in AD.

## 2. AMPK, Structure, and Function

AMPK is a key energy sensor, important for maintaining cellular energy homeostasis. It consists of three subunits: *α* (*α*1 and *α*2), *β* (*β*1 and *β*2), and *γ* (*γ*1, *γ*2, and *γ*3). The *α*-subunit is the catalytic unit and the others are regulatory. It is activated via allosteric binding of AMP to the *γ*-subunit or phosphorylation of the threonine residue 172 (Thr 172) of *α*-subunit [34]. Evidence shows that the main signals for its activation are the AMP/ATP ratio and level of cellular ROS [31].

Because we have different genes expressing each subunit, it is possible to have 12 AMPK complexes [35]. Several upstream kinases regulate the activity of AMPK, including the calmodulin-dependent protein kinase kinase (CaMKK), liver kinase B1 (LKB1), TGF-*β*-activated kinase 1 (TAK1), protein kinase A, and ataxia-telangiectasia mutated kinase (ATM) [34].

The major function of AMPK is to switch on energy-producing process and to switch off energy-consuming functions [35]. Thus, it promotes glucose uptake and glycolysis, fatty acid oxidation, and mitochondrial biogenesis which ultimately increase ATP production. On the other hand, it inhibits anabolic processes such as protein synthesis and decreases ATP consumption [35]. AMPK signaling also has a critical role in the hypothalamic neurons in regulating food intake [36].

There are emerging studies indicating that the function of AMPK is not restricted to the maintenance of energy metabolism during increased energy consumption, but it can coordinate to several housekeeping mechanisms, e.g., autophagocytosis of damaged structures and alleviate stress by increasing tissue stress resistance [35].

## 3. AMPK Role in Alzheimer's Disease

Despite the fact that AMPK usually has a positive effect on the health of an organism, there are certain shreds of evidence which show it can also be involved in the development of the disease. As highlighted in the introduction part, several studies have shown that activation of AMPK has a preventive role in AD [37, 38]. However, several other studies also reported that AMPK activation has an aggravating effect on the development of AD [39, 40]. Thus, the therapeutic potential of AMPK in AD remains to be controversial.

**Figure 1:**
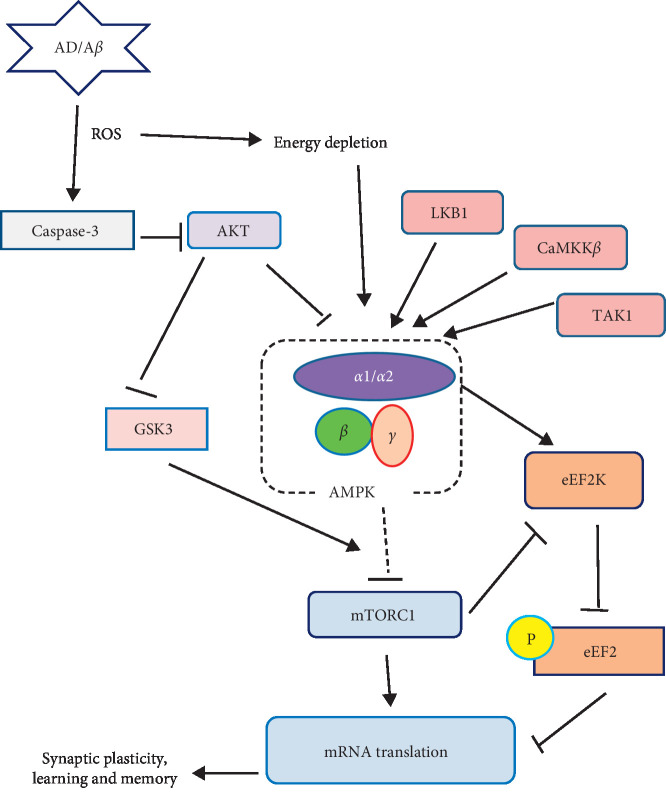
The AMPK signaling pathway: schematic model depicts the main upstream regulators and downstream effectors of AMPK. Potential link to AD is indicated. Arrows denote activation and blunted lines indicate inhibition [42].

AMPK appears to link energy metabolism to synaptic plasticity which in turn suggests energy deficiency is linked to an abnormality in synaptic transmission and memory impairment. Multifactor network analysis by Caberlotto et al. suggested that deregulation of various metabolic factors and energy homeostasis plays a key role in AD [41]. They also suggested that the AMPK is at the center of all these deregulations. This in turn explains the downstream alterations such as tangle formation, A*β* plaque formation, alterations in metabolic signaling and memory impairment, and inflammatory and apoptotic events [41].  Figure 1 summarizes the various effects of AMPK which impact the pathogenesis of AD (see Figure 1) [42]. In the succeeding portions, we review the effect of AMPK activation in AD with regard to its effect on amyloid-*β* aggregation, tau phosphorylation, and oxidative stress.

## 4. AMPK and A*β* Metabolism

Multiple lines of evidence have shown that overproduction of A*β* is associated with neuronal dysfunction and subsequent neuronal death. The fight against excessive cerebral accumulation of A*β* is one of the main objectives in clinical studies [43]. Amyloid-*β* peptides are produced by sequential cleavage of amyloid precursor protein (APP). In the amyloidogenic pathway of APP metabolism, the full length of APP is cleaved by *β*-secretase, BACE1. Then, the resulting C-terminal is cleaved by the *γ*-secretase, which generates A*β* [44–46]. Then, A*β* is cleared through different mechanisms including enzymatic degradation, glymphatic clearance, and autophagic lysosomal degradation, just to mention some [47].

There are controversial reports regarding the effect of AMPK activation on the metabolism of A*β*. Even those who reported the positive effect of AMPK on A*β* reported diverse mechanisms. Although there is controversy regarding the role of AMPK in AD development, there is no doubt about its involvement in the pathogenesis. Fewlass et al. showed that treatment with leptin decreases the accumulation of A*β* in Alzheimer's models and decreases the *β*-secretase activity and A*β* levels *in vitro* [48]. This was corroborated by epidemiological studies which showed a lower leptin level in AD patients as compared to healthy individuals [49, 50]. However, the mechanism of the leptin neuroprotective effect remained elusive.

After some years, Greco et al. demonstrated that leptin shows a similar effect with the aforementioned reports and this effect appears to be at least partly mediated by AMPK [51]. Those same authors also showed that direct stimulation of AMPK with 5-amino-imidazole-4-carboxamide ribonucleoside (AICAR) replicates leptins' effect and decreased A*β* production whereas AMPK inhibitor compound C increased A*β* production. Vingtdeux et al. also demonstrated that pharmacological activation of AMPK by resveratrol, a long known AMPK activator, lowers the extracellular concentration of A*β* [52].

In a study by Lu et al., they investigated the effect of quercetin, an AMPK activator, on amyloidogenesis and they found that the expression of BACE1 enzyme and A*β* generation was reduced by AMPK activation. The investigators concluded that AMPK activation decreases A*β* generation by decreasing the expression of BACE1, the rate-limiting enzyme in the generation of A*β* [53]. Hettich et al. also showed that metformin decreases BACE1 expression however by a mechanism that does not involve AMPK [54]. Recently, Zhang et al. also reported that activation of AMPK with berberine, and AICAR decreases the expression of BACE1 at the transcription and translation levels. This was evidenced by a decrease in the level of BACE1 in the western blot and decreased mRNA in the real-time PCR analyses. Consequently, they also showed a decrement in A*β* production in cultured cortical neuroblastoma cells [55].

With different AMPK activators, other studies also showed that BACE1 expression is reduced at the translation level as a result of AMPK activation [56, 57] and this was evidenced by attenuation of translation initiation factor eIF2*α* upon AMPK activation [56]. Moreover, as discussed below, activation of AMPK is known to activate autophagy and recent studies showed that upregulation of autophagy can reduce BACE1, which in turn can partially explain why the level of BACE1 is found to be reduced upon AMPK activation [58]. While it is evident that AMPK activates autophagy and autophagy decreases BACE1, the direct relationship between AMPK activation and BACE1 through autophagic process needs further exploration.

However, a study by Yang et al. brought a finding contrary to this, in which activation or inactivation of AMPK affects A*β* level without affecting the level of APP or the enzymatic activity of *α-* and *β*-secretases [59]. Another previous study also showed no effect of AMPK activation on BACE1 expression but decreased A*β* production [60]. Furthermore, MacPherson et al. also showed that reduction in the expression of BACE1 is accompanied by decreased activity of AMPK, contrary to the above studies [61]. Taking the above shreds of evidence together, AMPK activation decreases the generation of A*β*, but the effect on BACE1 expression remains to be controversial. Therefore, further studies are required to explore the effect of AMPK activators on the different transcription and translation factors and their exact effect on the expression of BACE1 enzyme.

Another study by Gupta et al. studied the effect of metformin, another well-known AMPK activator, on the metabolism of A*β* and showed that it reduces the brain level of A*β* [62]. The authors showed that metformin activates AMPK and reduces A*β* in cell lines; however, the effect of metformin through AMPK activation is just speculative. Interestingly, a recent study by Chen et al. clearly showed metformin reduces A*β* burden by AMPK-induced enhancement of autophagy. This was further consolidated by a genetic study in which neurons from AMPK*α*2 knockout mice showed increased A*β* generation [63].

Several other studies showed that AMPK activation reduces the A*β* burden by mechanisms other than involving BACE1. As highlighted above, resveratrol and metformin decrease the A*β* level by a mechanism related to enhanced autophagy [64].

Amplitude of evidence indicates that autophagy is highly impaired in AD patients [65, 66]. Although the mechanism of impairment remains unclear, the weight of evidence implicates the impairment mainly lies in the autophagosome maturation and clearance processes. This impairment in autophagic flux may be secondary to either impairment in axonal trafficking or insufficient lysosome acidification [67, 68]. Recent studies show that AMPK activation not only enhances autophagosome formation but also enhances autophagosome clearance. Recently, Jang et al. demonstrated that activation of AMPK activates autophagosome maturation and lysosomal fusion. The authors did the study on AMPK*α*1 knockout cell lines. Treatment of normal cells with compound C (AMPK-independent autophagy inducer) and trehalose (an mTOR-independent autophagy inducer) induced activation of autophagosomes and autolysosomes. On the other hand, treatment of AMPK*α*1 knockout cells with either of these compounds induced activation of autophagosomes but not autolysosomes. They also demonstrated that the decreased autolysosome formation is related to decreased autophagosome fusion with lysosomes in AMPK*α*1 knockout cells [69]. These shreds of evidence show that AMPK activation is important not only in autophagosome formation but also in autophagosome clearance and can improve the overall autophagic clearance of aberrant proteins. This was corroborated with many other pharmacological studies in which the role of AMPK in autophagic flux is demonstrated [63, 70, 71]. For instance, Chen et al. showed that AMPK activation with metformin enhances the autophagosomes clearance. They showed that AMPK-induced overall autophagy is inhibited by chloroquine, an autophagy inhibitor which works by inhibiting lysosomal acidification and degradation [71].

Importantly, several studies have shown the role of AMPK activators in autophagic clearance of A*β*. Recently, Zhao et al. showed that activation of AMPK by Radix Polygalae decreases A*β* by enhancing cellular autophagy [72]. They showed a dose-dependent increment in the phosphorylation level of AMPK and autophagy marker. These results are well consistent with recent studies by Park et al. in which activation of AMPK with cilostazol significantly increased the autophagic process and decreased the A*β* burden (see Table 1) [73]. Along this line, quite a recent study by Ou et al. demonstrated that metformin decreases the A*β* burden by activating autophagy which in turn is mediated by activation of AMPK which resulted in reduction of p-mTOR [78]. Together with the fact that enhanced autophagy promotes the clearance of A*β* [79–81] and AMPK is one of the major positive regulators of autophagy, a study by Salminen et al. [35] suggests AMPK activators can enhance the clearance of A*β* through induction of autophagy.

Moreover, recent studies show that autophagy has a role in the secretory pathway and is involved in the secretion of several proteins. One of them is an insulin-degrading enzyme (IDE), an enzyme highly involved in the degradation of extracellular A*β* [82, 83]. In AD pathology, astrocytes are the principal sources of IDE and can significantly degrade extracellular A*β* peptides, indicating that it may be important for AD progression. Li et al. demonstrated that AMPK activators increase the expression of IDE in astrocytes [74]. The authors observed that animal models of AD and T2DM are found to have decreased level of IDE and treatment with AMPK activators improved the IDE expression and lowered the level of A*β*. Previous study by Son et al. also brought similar report in which they showed activation of AMPK with simvastatin induces extracellular IDE secretion from astrocytes via an autophagy-based unconventional secretory pathway and resulted in reduction of A*β* level. This was evidenced by the fact that the antiamyloidogenic effect of simvastatin was reversed with compound C (AMPK inhibitor), 3-MA (autophagy inhibitor), and bacitracin (IDE inhibitor) [75]. This shows that activation of AMPK induces autophagy and induced autophagy leads to increased production of IDE. This could result in a synergistic clearance of A*β*.

Another reported mechanism by which AMPK regulates amyloidogenesis is by regulating the metabolism of cholesterol and sphingolipid. AMPK regulates cholesterol and sphingolipid biosynthesis by regulating gene expressions and activities of associated enzymes. Won et al. implicated that production of cholesterol and sphingolipids is reduced by AMPK activators such as AICAR. On the contrary, genetic deletion of AMPK*α*2 significantly increases the generation of both lipids [37]. Studies show that amyloidogenic enzymes (BACE1 and *γ*-secretase) are found on the lipid rafts of the cell membrane which is critical for their role in the production of A*β* [84, 85].

On the other hand, the APP is found on both the lipid rafts and nonlipid raft fractions [86]. Treatment with AMPK activators selectively inhibits the distribution of APP in the lipid rafts without affecting BACE. This in turn decreases the production of A*β*. Won et al. demonstrated that this selective redistribution of APP by AMPK activators is due to their effect on the production of sphingolipids. Interestingly, AMPKa2 KO neurons showed an opposite effect to AICAR-treated neurons [37]. Recent pieces of evidence also show that reduction of cholesterol and sphingolipids decreases the generation of A*β* [87, 88]. However, the exact effect of AMPK activators on A*β* through sphingolipids and cholesterol metabolism needs direct investigation as there are many other reported mechanisms by which AMPK activators can decrease A*β* metabolism.

Combining all the above shreds of evidence, AMPK activation by different drugs decreases the generation and accumulation of A*β* through mechanisms including improved autophagy, increased IDE expression, decreased expression of BACE1, and decreased production of cholesterol and sphingolipids.

On the contrary, negative effects of AMPK activation on A*β* metabolism were also reported. A population-based large-scale case-control study showed that the treatment of T2DM patients with metformin showed a slightly higher risk of AD development [89]. This was against several epidemiological studies which showed a lower risk of AD in metformin-treated individuals [39, 40, 90]. However, there are *in vitro* and animal studies which also show a negative effect of metformin and support the argument that A*β* generation is increased after treatment with AMPK activators (see Table 1). Chen et al. investigated the effect of metformin on the secretion of A*β* and they found increased A*β* by increasing the expression of BACE1 at transcription level which was evidenced with increased level of BACE1 and BACE1 mRNA [91]. This was furthered by Son et al. in which they demonstrated metformin increases the generation of A*β* but with a different mechanism. The authors revealed that metformin treatment of SH-SY5Y cells causes accumulation of autophagosomes resulting in an increased *β*- and *γ*-secretases and A*β* generation (see Table 1) [76]. Moreover, Picone et al. also studied the biological effects of AMPK activator metformin on the generation of A*β* and expression of APP and BACE1 and they found that AMPK activation increases A*β*, BACE1, and APP expression [77]. Therefore, there is prevalent evidence that activation of AMPK with metformin plays a negative role in the pathogenesis of AD.

However, as mentioned above, the evidence is so diverse and conflicting. The epidemiological data differences can be attributed to the differences in the protocols used, duration of treatment, presence of comorbidities, and concomitant medication and other confounding factors as most of the studies did not address the confounding factors. In the animal studies, the controversial findings may arise from differences in the protocols/culture differences, differences in the type of model used in animal models, the presence of insulin, and the mechanism of AMPK activation. Therefore, randomized clinical studies are warranted to reveal the clinical effects of AMPK activation on the risk of AD. Preclinical studies on different animal models and standard protocols are also needed to explore the exact effect of AMPK activators on A*β* accumulation and aggregation. As a matter of fact, no animal model recapitulates the entirety of human AD pathophysiology [92]. Additionally, the stress responsivity of the different AD mouse models is reported to be highly variable [93]. Thus, the limitations of every animal model used should be clearly characterized as some of the conflicting findings can be attributed to these limitations.

Moreover, many of the conflicting effects reported came from studies on metformin. Intraperitoneal versus intraventricular administration of the drug has shown a diverging effect in animal models of stroke; intraperitoneal administration showed neuroprotective effect and intraventricular administration increased neuronal damage [94]. In addition, the cerebrospinal fluid concentration of metformin achieved from peripheral administration is 4% compared to the plasma concentration if the blood-brain barrier is not inflamed [95]. These couple of evidences show that metformin affects the AD pathology by peripheral mechanisms. Therefore, further study on this specific drug should focus on the differential effect of central and peripheral administration on A*β* metabolism.

## 5. AMPK and Tauopathy

At physiological conditions, tau proteins are soluble microtubule-associated proteins sorted into neuronal axons. Under abnormal conditions, tau protein undergoes many alterations which are thought to impair its function and undergo neurodegeneration [96, 97]. These alterations are regarded as posttranslational modifications which include phosphorylation, acetylation, and ubiquitination among the many others. The phosphorylation of Ser/Thr residues is the main modification, which regulates the binding of tau to the tubulin protein in microtubules. Abnormal hyperphosphorylation of tau can trigger the self-assembly of tau proteins into paired helical filaments (PHFs) and subsequently induce the fibrillization of PHFs into deposits of neurofibrillary tangles and ultimately cause a tauopathy [35, 97].

Intracellular aggregates of these hyperphosphorylated tau proteins are regarded as neurofibrillary tangles and are a hallmark of AD together with A*β* [35]. Although the exact cause of changing tau condition from balanced phosphorylation to hyperphosphorylation is not clearly understood, some recent studies implicate metabolic imbalance involving AMPK to play a role in tau pathology [98–100].

Tau protein phosphorylation at different sites is mediated by proline-directed tau kinases (e.g., GSK3*β*, Cdk5, and ERK) and non-proline-directed tau kinases such as protein kinase, CaMKII, AMPK, and MARK1. Of the different phosphorylation sites, phosphorylation at Ser_396_ residues results in a reduction in microtubule function [101, 102]. Phosphorylation at this site is mediated by different kinases, but it is reported that GSK3*β* is the major tau kinase [103]. In addition, the level of hyperphosphorylation is regulated by the balance between phosphorylation by the different kinases and activity of dephosphorylating enzymes phosphatases mainly protein phosphatases 2A (PP2A) [104].

Although phosphorylation, received initial attention as the primary event for the tau aggregation process, recently acetylation of tau protein has also emerged to be one of the posttranslational modifications of tau protein which can regulate tauopathy [105, 106]. Acetylation is found to reduce proteasomal degradation of tau protein, inhibit tau microtubule binding, and promote tau aggregation [106–109]. Interestingly, several proteins are involved in the acetylation and deacetylation of tau protein [110–112]. The most important antiaggregative deacetylation is NAD^+^-dependent sirtuin 1 deacetylase (SIRT1) [110, 113]. Mounting evidence shows that AMPK enhances SIRT1 activity by increasing the availability of NAD^+^ [114]. The effect of AMPK on SIRT1 and tau acetylation is discussed below.

There are controversial data regarding the effect of AMPK activation on tauopathy. Given the fact that AMPK is a known autophagy activator [115–117] and autophagy-lysosomal system is the main mechanism of aggregated tau clearance [118–120], it is possible to speculate from the outset that AMPK activation has an antitauopathy effect. However, the effects of AMPK are so diverse and results from *in vivo* and *in vitro* studies show contentious effects. The amyloid hypothesis states that high level of A*β* induces the posttranslational tau modifications which are protauopathy. In fact, the deleterious effects of A*β* on neuronal degeneration depend on tauopathy in large part. However, as mentioned above, the effect of AMPK on A*β* also remains controversial. Thus, the effect of AMPK activation on tauopathy through reduction of A*β* needs direct evaluation. In addition, tau misaggregation also occurs independently of A*β* [121]. Moreover, there are conflicting reports regarding the effect of A*β* on AMPK; some reports say A*β* stimulates AMPK [122, 123] and some others say the effect is inhibitory [124–127]. Therefore, before making these speculations, two things require clarification: (i) the exact effect of A*β* on AMPK whether it is stimulatory or inhibitory; and (ii) the effect AMPK on tau phosphorylation via its downstream molecules should be explored. The second issue is addressed below.

It has been shown that tau phosphorylation is significantly increased in animal models of T2DM, where there is impairment in AMPK [128] and previous studies suggested that this increased tau phosphorylation could be related to inhibition of PP2A, decreased SIRT 1 activity, and overactivity of GSK3*β* [92, 104, 129, 130]. Interestingly, several studies have shown that activation of AMPK mitigates tauopathy by different mechanisms including (1) decreasing the activity of GSK3*β*, (2) increasing the activity of phosphatases, particularly PP2A, and (3) enhancing proteasomal degradation by activating SIRT1 deacetylation [110, 113, 131, 132]. The succeeding paragraphs discuss the current evidence on how AMPK affects these downstream players and their effect on tauopathy.

Starting with the first mechanism, Greco et al. studied the effect of leptin on tau phosphorylation induced by retinoic acid in human cell lines: SH-SY5Y and NTera-2, and they revealed that it reduces the phosphorylation of tau proteins at AD-specific amino acid residues. They also observed that AICAR mimics the effect of leptin, and on the other hand, compound C antagonizes the effect of leptin [51]. Subsequently, Greco et al. investigated the effect of AICAR on tau pathology and the downstream mechanism and they found that it reduces the formation of hyperphosphorylated tau by inhibiting GSK-3*β* [131]. Similarly, using different AMPK activators, Kim et al., Park et al., and Kornelius et al. also showed that AMPK activation decreases tau hyperphosphorylation by inhibiting GSK-3*β* [38, 127, 133]. In these studies, the antitauopathy effect through GSK-3*β* inhibition was clearly implicated as the effects were reversed by compound C.

Using senescence-accelerated P8 mice (SAMP8) model, a well-known AD model, Kim et al. investigated the role of AMPK in tau phosphorylation. The investigators found that AMPK activation was significantly higher than the controls, which was concurrent with lower p-tau expression. Importantly, the investigators found that GSK3*β* was significantly phosphorylated at a site which turns it to an inactive form. The authors concluded that overexpression of AMPK observed in the SAMP8 mice could be a compensatory response to the derangements in the metabolic machinery and to reverse tauopathy and other pathologic processes leading to memory impairment. They also studied the effect of AICAR *in vitro* on differentiated SH-SY5Y cells and they reported that it significantly reduces p-tau whereas compound C enhances tau phosphorylation. Furthermore, their findings suggested that AMPK-mediated decrement in tau phosphorylation is at least partly caused by inhibition of GSK3*β* as a significant increase in the inhibitory phosphorylation of GSK3*β* was observed in the AICAR-treated SH-SY5Y cells. This was furthered by Kim et al. using the same model in which the authors reported increment in the phosphorylation of tau and activation of AMPK-GSK3*β* pathway in the cortex of SAMP8. Taking the above evidence together, it is strongly evidenced that AMPK regulates tau phosphorylation by inhibiting GSK 3*β* [92].

The second mechanism by which AMPK activation is found to reduce tau phosphorylation is by activation of PP2A [104]. As described above, PP2A is one of the different enzymes involved in the regulation of tau phosphorylation. Several studies have shown that the level and activity of PP2A are found to be lowered in AD animal models and in postmortem AD patients, showing its strong involvement in the generation of tauopathy [134–136]. In addition, the use of PP2A blocker, okadaic, elevates the phosphorylation of tau protein in cultured cells and animal models [137, 138]. Interestingly, a handful of evidence shows that PP2A activity is regulated by AMPK; i.e., PP2A activity is increased by AMPK-mediated phosphorylation at its regulatory Ser^298^ and Ser^336^ amino acid residues [104, 139, 140]. AMPK activation upon nutrient starvation and carbon depletion is also found to result in an increased expression of PP2A [141]. Collectively, the above pieces of evidence show that PP2A is strongly involved in the pathogenesis of tauopathy and its function is regulated by AMPK.

Many studies involving AMPK activators are found to increase the level and activity of PP2A. Kickstein et al. demonstrated that metformin enhances PP2A activity and decreases tau phosphorylation at PP2A-dependent epitopes in an *in vivo* and *in vitro* models but in an AMPK-independent mechanism [142]. Likewise, Pérez-Revuelta et al. showed that metformin activates AMPK, and then AMPK inhibits mTOR which then leads to activation of PP2A [143]. This is in line with previous studies which showed that inhibition of mTOR by rapamycin activates PP2A [144]. Moreover, AMPK is widely recognized as mTOR antagonist [145]. However, the authors did not directly ascribe the antitauopathy effect of the drug to AMPK activation. Therefore, the antitauopathy effect of metformin through AMPK-mediated PP2A activation is just speculative. Recent study however showed the direct effect of AMPK activation on PP2A activation and tau dephosphorylation. Kim et al. showed the antitauopathy effect of AMPK activation. They did the study on the SAMP8 mice model and they confirmed that AMPK activation increases PP2A activity. In an *in vitro* study, those same authors showed that okadaic, PP2A antagonist, and compound C reversed the effect of AICAR on tau phosphorylation. This shows that PP2A mediates the antitauopathy and neuroprotective effect of AMPK activators Therefore, PP2A might be another AMPK downstream player mediating the antitauopathy and neuroprotective effects of AMPK activators. Indeed, PP2A is a highly studied novel target for tauopathies including AD, and there are drugs which are under clinical trials [97]. Nevertheless, the evidence showing PP2A mediating the antitauopathy effects of AMPK activators is so scarce, and more studies are required to uncover the extent of the AMPK effect on PP2A [104].

Third mechanism by which AMPK decreases tau phosphorylation and misaggregation is by activating SIRT1. As mentioned above, tau acetylation decreases its degradation. Studies show that acetylated tau proteins are found to be increased in AD patients and their appearance precedes the hyperphosphorylation and tangle formation. This increased acetylation level is attributed to the decreased level of SIRT1 level [110]. This is evidenced by the following: (1) SIRT1 is found to abolish the acetylation of tau in vitro [110] and (2) decreased level of SIRT1 was found in AD patients which was correlated with tau levels [104, 113]. Moreover, a good number of studies showed that pharmacological activation of SIRT1 enhances the clearance of misfolded tau through the ubiquitin-proteasomal system [132, 146]. Interestingly, a substantial body of evidence shows that AMPK is a potent activator of SIRT1 [114, 147]. Moreover, recent study has shown that fisetin (herbal product) through activation of AMPK-SIRT1 pathway reduced tau phosphorylation as it was evidenced with decreased p-tau and increased p-AMPK and SIRT1 in western blot. Therefore, activation of AMPK can increase the clearance of misfolded tau proteins by activating its downstream actor, SIRT1 [148]. Again, this should be further confirmed using AMPK KO models or AMPK inhibitors.

As mentioned in the preceding parts, tau hyperphosphorylation occurs when there is disequilibrium between tau kinases and phosphatase activities (mainly an imbalance between GSK3*β* and PP2A enzyme activities). Looking at the effect of AMPK on GSK3*β* and PP2A and their respective effects on tau phosphorylation separately, one might conclude that the net effect of AMPK is double-edged sword having an additive effect on the tau dephosphorylation. However, the effect of AMPK on its downstream actors is hellishly complicated and it is not straightforward to make a conclusion about the net effect on tau phosphorylation. For example, PP2A and GSK3*β* are not only common targets for AMPK, but also there is interplay between them. On the one hand, PP2A dephosphorylates tau protein directly, opposing the effect of GSK3*β*, and on the other hand, PP2A dephosphorylates upstream inhibitors of GSK3*β* which leaves it active [149–151]. In addition, PP2A removes the phosphate group from the inhibitory site of GSK3*β* turning it to the active form. But it is not only one-way street; GSK3*β* also inhibits the activity of PP2A [151, 152]. Therefore, the net effect of the interplay between these actors is confusing and needs further clarification. Further complicating the matter, the interaction of AMPK with PP2A and GSK3*β* is not only one-way street; these downstream molecules also negatively regulate the activity of AMPK. It is evident that GSK3*β* inhibits the activity of AMPK [153]. This backward effect produces antitauopathy effect because AMPK inhibits GSK3*β* and GSK3*β* inhibition activates AMPK. Therefore, if the positive reports of AMPK on tauopathy are true, the net effect of this interplay is positive, decreasing tauopathy.

On the other hand, several studies have shown that PP2A inactivates AMPK and this interplay may produce a negative effect on tauopathy [154, 155]. Therefore, future studies on the effect of AMPK on tauopathy should take this complex interplay into account in order to get a full picture of the AMPK effect on tau pathogenesis.

Like the effect on A*β*, negative effects of AMPK activation on tauopathy were also reported by several studies. Thornton et al., Vingtdeux et al., and Mairet-Coello et al. have shown that recombinant activated AMPK phosphorylates tau proteins at several sites in the microtubule-binding domains [122, 123, 156]. Vingtdeux et al. demonstrated that phosphorylated AMPK is highly accumulated in the cytoplasm of cerebral neurons of AD as opposed to the normal neurons in which phosphorylated AMPK is localized to the nucleus. Likewise, Thornton et al. also demonstrated that AMPK is rapidly activated after exposure to A*β* and they found it to be a tau kinase [122]. Mairet-Coello et al. also showed similar findings and were associated with dendritic spine loss. Moreover, Domise et al. studied the effect of pharmacological AMPK activators (AICAR and metformin) on tau phosphorylation in primary differentiated neurons and they observed that treatment of the cells with either of the two drugs for 24 hours significantly increased the phosphorylation of tau proteins. They also showed that the activated AMPK and phosphorylated tau were colocalized and the phosphorylations were at sites relevant to affect the microtubule binding as the authors observed decreased microtubule binding in drug-treated cells [157]. Interestingly, the authors also reported that treatment with AMPK inhibitor, compound C, reversed the effect of AMPK activators. These findings were recapitulated with in vivo studies. The same authors studied the level of tau phosphorylation on AMPK*α*2 KO and AMPK*α*2 KO:tauP301S mice and they found that tau phosphorylation was lower than that observed in AMPK*α*2+/+:tauP301 [157]. Taking these shreds of evidence together, AMPK is a direct tau kinase and mediates the detrimental effects of A*β*. However, as tau protein can be phosphorylated at more than 40 sites [158], the effect of the phosphorylation at AMPK sensitive epitope on memory and learning needs extensive exploration.

One previous study reported that AMPK-induced tau phosphorylation alone is not associated with learning and memory loss, which suggests cophosphorylation at additional sites by different other kinases is required [158]. The findings also suggested that the presence of PP2A and GSK3*β* dysfunction is required for memory and learning impairments. As discussed above, PP2A activation and GSK3*β* inhibition mediate the antitauopathy effects of AMPK. Therefore, the net effect of direct AMPK-mediated tau phosphorylation and indirect PP2A and GSK3*β*-mediated dephosphorylation deserves further research.

All in all, the net effect of AMPK activators in tau phosphorylation remains to be clearly established. As already described, AMPK has a direct negative effect on tauopathy and has an indirect protective effect through its downstream mediators. In addition, the indirect positive effects of AMPK are complex as there is bidirectional regulation of the different regulators of tau phosphorylation. Therefore, a detailed study involving different molecules involved in the regulation of AMPK and downstream to AMPK is warranted to establish the exact effect AMPK on tauopathy.

## 6. AMPK and Oxidative Stress

Oxidative stress is defined as a redox state that occurs due to an imbalance between generation and detoxification of reactive radicals in the body, which can lead to cell and tissue damage. It is one of the main causes of AD and it is one of the key hypotheses [159, 160].

Multiple studies have shown that oxidative stress is an early response in AD patients and AD animal models and occurs prior to A*β* accumulation. This is consistent with the findings that oxidative stress stimulates the enzyme BACE1 and enhances A*β* production [161–164]. This suggests that A*β* is an epiphenomenon of pathological development of AD rather than the cause of the disease. Thus, oxidative stress plays a central role in the pathogenesis of AD [165–171]. The fact that oxidative stress is a common feature of the pathophysiology of neurodegenerative diseases also supports the above notion [172]. However, A*β* once produced also contributes to the oxidative damage by enhancing the production of reactive species due to damage to the mitochondria [169, 173, 174]. Thus, vicious cycle may ensue once mitochondrial dysfunction occurs, i.e., oxidative stress facilitates the production of A*β*, and A*β* in turn is toxic to the mitochondria and causes further oxidative damage.

There is also a close interplay between oxidative stress and tauopathy. Although “which one appears first” remains far from clear, it is almost established fact that oxidative stress and tauopathy are key components of vicious circle playing a crucial role in the pathogenesis of AD [175–179]. Several lines of evidence showed that oxidative stress is the consequence of cellular damages caused by tauopathy [180–182]. However, mounting recent evidence strongly suggests that oxidative stress directly promotes the phosphorylation of tau, and thus, tauopathy is consequential to oxidative stress [172]. Although “which comes first?” remains an open question, it clear that the hallmarks of AD (tauopathy, amyloid plaque, and oxidative stress) are pathologically interconnected and are components of the vicious circle. Interestingly, it seems that their commonalties lie down to the mitochondrial function [174].

Mitochondria are the major professional ROS producers through their central role in energy metabolism. Mitochondrial dysfunction is thus implicated the most in oxidative stress. Normally, mitochondria permanently produce ROS as a by-product of ATP. However, a pool of antioxidants is also produced to detoxify the ROS. Therefore, in normal people, there is a delicate balance between ROS and antioxidants. Oxidative stress occurs when there is an imbalance between prooxidants and antioxidant level in the brain cells. Importantly, after perturbations to the redox balance, the vicious circle of oxidative damage ensues [183–187].

Neuronal cells are particularly susceptible to oxidative stress because the redox balance is highly fragile [183, 188]. Thus, several aspects of mitochondria can be exploited as promising targets for therapeutic development for AD. Dysregulation of AMPK is strongly associated with oxidative stress as it regulates various aspects of mitochondrial function and can be targeted for therapeutic inroads [31].

AMPK is a redox-sensitive protein. Amplitude of evidence shows that AMPK is activated in response to excess ROS states. Thus, it couples the cellular ROS level to mitochondrial metabolic homeostasis. For instance, Rabinovitch et al. demonstrated that treatment of mice embryonic fibroblasts with vitamin E analogue (antioxidant) reduced the basal activation of AMPK. This shows that AMPK is activated in response to cellular ROS excess [189]. On the other hand, AMPK regulates various aspects of mitochondrial biology and homeostasis including stimulation of mitochondrial biogenesis, mitochondrial quality control, and regulation of mitochondrial shape in cells [31].

AMPK regulates mitochondrial ROS level. This is evidenced by several studies. Rabinovitch et al. demonstrated that cell lines lacking AMPK*α* expression displayed 50% higher basal levels of mitochondrial ROS, compared to normal cells. Consistent with this, the AMPK*α* knockout cells also rapidly underwent senescence (the hallmark of oxidative stress) compared to control cells [189]. Another study by Vincent et al. also brought consistent finding in which they showed that treating cells with A-769662 (AMPK agonist) blocked mitochondrial ROS production induced by glucose withdrawal [190]. Several other AMPK activators such as metformin, resveratrol, and AICAR are also reported to decrease intracellular and mitochondrial ROS burden. These drugs reversed lipid peroxidation and enhanced antioxidant contents such as SOD, catalase, and GSH [191–194].

Different mechanisms are reported for the antioxidant function of AMPK. AMPK modulates the activity of several downstream proteins and processes involved in the redox balance. Some of the antioxidant mechanisms are discussed in the succeeding paragraphs, and we grouped them into four broad mechanisms: (1) enhancing the expression of antioxidants, (2) activating antioxidant enzymes, (3) decreasing the generation of ROS, and (4) stimulating autophagic clearance.

The first mechanism is by stimulating the expression of antioxidant genes and it involves different pathways. AMPK is the upstream activator of nuclear factor erythroid 2-related factor 2 (Nrf2), a master regulator of antioxidant gen programs [195–197]. Once activated, Nrf2 translocates to the nucleus where it binds with the antioxidant response element and stimulates the expression of different antioxidant proteins such as heme oxygenase-1, NADPH quinone oxidoreductase-1, and superoxide dismutase [198–200]. Nrf2 is found to be reduced in the nucleus of hippocampal neurons of AD patients and APP/PS1 transgenic mice [201, 202]. In addition, hippocampal injection of Nrf2 improves spatial learning in APP/PS1 transgenic mice [203]. Moreover, Nrf2 activators such as curcumin display antioxidant activity [204]. These shreds of evidence in concert indicate that Nrf2 is strongly implicated in antioxidative stress. Interestingly, several studies have shown that AMPK is a strong upstream activator of Nrf2 [205, 206]. This was clearly shown in *in vivo* and *in vitro* studies in which the neuroprotective effect of AMPK activators was reversed by genetic deletion of both AMPK and Nrf2 genes. Therefore, AMPK/Nrf2/antioxidant enzyme pathway is one antioxidant mechanism of AMPK activators.

There is also another downstream actor which enhances the AMPK-mediated expression of antioxidant enzymes, PGC-1*α*. Similar to Nrf2, once activated by AMPK, PGC-1*α* translocates to the nucleus and stimulates the expression of superoxide dismutase, catalase, and glutathione peroxidase-1 [207, 208].

The second mechanism involved in the AMPK-dependent enhanced antioxidant effect is the activation of the AMPK-SIRT3-SOD2 pathway. This pathway activates the already synthesized antioxidants. SIRT3, a NAD^+^-dependent protein deacetylase, is highly localized in the mitochondria [209]. Multiple lines of evidence show that SIRT3 is highly involved in AD pathogenesis. This is evidenced by fact that SIRT3 levels are found to be reduced in cortical neurons of AD [210, 211], and overexpression of SIRT3 enhances the oxidative resistance of hippocampal neuronal cultures [212]. As reviewed recently by Khanna et al. [209], activation of AMPK enhances the expression of SIRT3. SIRT3 in turn regulates the activity of enzymatic antioxidants by regulating the acetylation (deacetylation turns it to an active form). SIRT3 particularly deacetylates SOD2 and decreases mitochondrial ROS [213, 214]. Thus, AMP/SIRT3/SOD2 pathway serves as an antioxidant cascade by enhancing the expression of SIRT3 which in turn activates SOD2.

A third reported mechanism is through inhibition of the generation of ROS by inhibiting NADPH oxidase. NADPH oxidase is one of the major sources of ROS in the brain [215]. Ceolotto et al. investigated the effect of AICAR and rosiglitazone, on human umbilical vein endothelial cells treated with high glucose. They observed that treatment with either AICAR or rosiglitazone decreased the generation of ROS. Interestingly, they showed that these drugs decreased the expression of NADPH oxidase, which underscores the involvement of this enzyme in the antioxidant effect of these drugs. Silencing AMPK with siRNA or cotreatment with compound C reversed the effect of these drugs. Collectively, these pieces of evidence show that the antioxidant effect of rosiglitazone and AICAR is mediated by AMPK-dependent inhibition of NADPH oxidase [216]. This was furthered by different other studies using resveratrol [194, 217, 218]. Thus, AMPK-dependent activation of NADPH oxidase and consequent reduction of ROS generation play an immense role in the antioxidant effect of AMPK activators.

The fourth mechanism is by stimulation of autophagy. It is an established fact that one of the major actions of AMPK is mediating autophagy. Interestingly, autophagy is activated during break-in cellular homeostasis [219]. Body of shreds of evidence shows that autophagy, in turn, regulates the redox status by at least two mechanisms. These mechanisms are (i) selective engulfing and degradation of oxidized substances [219] and (ii) activation of antioxidant responses driven by the autophagy receptor p62/SQSTM1 [220]. Given that AMPK is the genuine energy and redox sensor, it can be said that AMPK activators have antioxidant activity mediated by autophagy.

In summary, AMPK signaling is an important system for sensing and resolving mitochondrial redox imbalances and maintaining metabolic balance. As described above, activated AMPK reduces ROS by several mechanisms. This redundant antioxidant effect of AMPK makes it an attractive target for oxidative stress in AD. Furthermore, the main problem with ordinary antioxidant treatment is their limited distribution to the mitochondria [221]. In light of this, AMPK is an attractive target for the mitochondrial-mediated treatment of AD.

## 7. The Pros and Cons of AMPK Activators in AD

Most of the AMPK activators used in most of the studies included in this review are indirect activators which work by increasing cellular AMP/(ADP): ATP ratio. Therefore, it is not always clear whether the effects of these agents are mediated by AMPK. In addition, most of these agents have shown AMPK-independent actions, which are also clearly ascribed to their neuroprotective effect in AD. For instance, as extensively reviewed by Ahmed et al. and Gomes et al., resveratrol prevents oxidative stress and neuroinflammation by activating SIRT1, inhibits the tauopathy by interfering with the midline 1-protein phosphatase 2A complex, improves learning and long-term memory formation by enhancing BDNF expression, and prevents the neuronal cell death by attenuating apoptosis via Akt/p38 MAPK signaling, to mention among the many other mechanisms [222, 223]. Similarly, metformin has also demonstrated several AMPK-independent neuroprotective mechanisms in AD. It has antioxidative and anti-inflammatory action mediated by activating SIRT1 [224], inhibits acetylcholine-esterase enzyme and enhances cholinergic neurotransmission, and reduces tau phosphorylation by activating PP2A independent of AMPK. Therefore, given that most drugs are indirect AMPK activators and most of them have multiple targets with a role in AD, it is important that future studies on the role of AMPK activation AD focus on the specific direct AMPK activators as some of the conflicting results may arise from the diversity of additional molecular targets.

As the review focuses on AMPK activation's effect in AD pathogenesis, here we summarized the pros and cons of AMPK activation. As mentioned in the previous section, the evidence on the AMPK effect in oxidative stress is consistent and it is protective. In contrast, the effect of AMPK activation in amyloid metabolism and tauopathy is controversial and both positive and negative effects have been reported. The following table summarizes the protective and aggressive effects of AMPK activators in tauopathy, *β*-amyloidopathy, and oxidative stress (see Table 2).

## 8. Conclusion

A great deal of knowledge about AMPK function has been accumulated over the years and brought the identification of several AMPK modulators. Considering all its downstream actors and their physiological effects, disturbances in AMPK signaling may explain many of the cellular abnormalities and functional disturbances of AD. However, the effect of AMPK activators in AD remains contentious. A good number of studies reported that AMPK activation reduces plaque and tangle formation and oxidative stress. But on the contrary, substantial amount evidence also showed the detrimental effect of AMPK activators. Therefore, further investigations are required to elucidate the exact relationship between AMPK and AD. Future studies should focus on exploring the possible effect differences among the different AMPK activators, AMPK subtype response differences for AMPK activators, and the extent of the involvement of AMPK in regulating the function of PP2A, GSK3*β*, and SIRT1.

## Figures and Tables

**Table 1:** The effects of AMPK activators on *β*-amyloidopathy.

AD biomodel	AMPK activator used	Main actions of AMPK activator observed	Mechanism of anti- or pro-A*β* effect	Ref.
High-cholesterol-fed old mice	Quercetin	(i) Quercetin significantly activated AMPK, reduced BACE1 enzyme, lowered A*β* deposit, and improved behavioral performance (ii) The neuroprotective effects were reversed with compound C (CC) (AMPK inhibitor)	Decrement in BACE1 enzyme expression	[53]

N2a/APP695sw cell lines	Berberine	(i) Reduced BACE1 expression and A*β* deposit (ii) AMPK activation increased (iii) Action reversed with compound CC	Decrement in BACE1 enzyme expression	[55]

APP/PS1 transgenic mice and HEK293 and N2a cell lines	Resveratrol	(i) Reduced A*β* deposition both *in vitro* and *in vivo* (ii) Genetic activation of AMPK reduced A*β* whereas genetic inhibition of AMPK increased A*β* (iii) Autophagy inhibitor, chloroquine, increased A*β* (iv) Effects of resveratrol were reversed with CC	Activation of autophagic processes	[52]

db/db mice and high glucose-cultured HT22 cells	Metformin	(i) Increased AMPK activation and reduced A*β* & p-tau (ii) Increased autophagy marker, LC3-II (iii) Effect was reversed with CC and chloroquine (autophagy inhibitor) (iv) Genetic suppression of autophagy reversed the effect of metformin	Activation of autophagic processes	[71]

N2aSwe cells	Cilostazol	(i) Increased P-AMPK and reduced A*β* (ii) Enhanced autophagic marker (iii) Effects were reversed with CC and bafilomycin A1 (autophagy inhibitor)	Activation of autophagic processes	[73]

A*β*PPswe/PSEN1dE9 mice model	AICAR and rosiglitazone	(i) AMPK and IDE downregulated and A*β* increased in the untreated A*β*PPswe/PSEN1dE9 mice (ii) AICAR and RSZ increased IDE expression decreased A*β* level	Increase in the expression of IDE	[74]

Astrocytes from newborn mice	Simvastatin	(i) Increased IDE expression and A*β* degradation (ii) Bacitracin A, an IDE inhibitor, reversed the effect on A*β* (iii) 3-MA (autophagy inhibitor) and CC reversed the effect of simvastatin on IDE level	Increase IDE expression by autophagy-based secretory pathway	[75]

SH-SY5Y cell lines and Tg6799 AD mice model	Metformin	(i) Increased *β*- & *γ*-secretase cleavage of APP and A*β* deposit (ii) Abnormal autophagic vacuole accumulation (iii) Effect was fully reversed with CC but partially reversed with 3-MA	Excessive autophagy mediated increase A*β* level	[76]

N2a695 cells	Metformin	(i) Increased BACE1 expression and A*β* generation (ii) BACE1 mRNA increased (iii) Effect was reversed with CC	AMPK activates the transcription of BACE1 mRNA	[77]

**Table 2:** Summary of the positive and negative effects of AMPK activators.

Positive effects	Negative effects
Inhibit BACE enzyme expression at translation level [53, 55]	Enhance BACE mRNA transcription [77]
Enhance autophagic clearance of A*β* and aggregated tau [71, 73]	Excessive autophagy-mediated secretion of A*β* [76]
Increased IDE expression and A*β* degradation [75]	AMPK directly phosphorylates tau proteins at sites which affect microtubule association [157]
Decrease the activity of GSK3*β* and thereby decrease tau phosphorylation [104, 225]	
Increase the activity of PP2A and facilitate the dephosphorylation of tau proteins [104]	
Activate SIRT1 and facilitate tau clearance through ubiquitin-proteasomal system [114, 148]	
Halt oxidative stress by reducing ROS production, activating antioxidant enzymes, enhancing the expression of antioxidants, and stimulating autophagic clearance [190, 205, 214, 219]	
